# The Blood–Brain Barrier, an Evolving Concept Based on Technological Advances and Cell–Cell Communications

**DOI:** 10.3390/cells11010133

**Published:** 2021-12-31

**Authors:** Camille Menaceur, Fabien Gosselet, Laurence Fenart, Julien Saint-Pol

**Affiliations:** University Artois, UR 2465, Laboratoire de la Barrière Hémato-Encéphalique (LBHE), F-62300 Lens, France; camille.menaceur@univ-artois.fr (C.M.); fabien.gosselet@univ-artois.fr (F.G.); laurence.tilloy@univ-artois.fr (L.F.)

**Keywords:** blood–brain barrier, historical description, development, maintenance, cell–cell communication

## Abstract

The construction of the blood–brain barrier (BBB), which is a natural barrier for maintaining brain homeostasis, is the result of a meticulous organisation in space and time of cell–cell communication processes between the endothelial cells that carry the BBB phenotype, the brain pericytes, the glial cells (mainly the astrocytes), and the neurons. The importance of these communications for the establishment, maturation and maintenance of this unique phenotype had already been suggested in the pioneering work to identify and demonstrate the BBB. As for the history of the BBB, the evolution of analytical techniques has allowed knowledge to evolve on the cell–cell communication pathways involved, as well as on the role played by the cells constituting the neurovascular unit in the maintenance of the BBB phenotype, and more particularly the brain pericytes. This review summarises the key points of the history of the BBB, from its origin to the current knowledge of its physiology, as well as the cell–cell communication pathways identified so far during its development, maintenance, and pathophysiological alteration.

## 1. Introduction

The blood–brain barrier (BBB) is a natural barrier that is crucial for maintaining brain homeostasis. This barrier isolates the brain from the bloodstream and regulates the bidirectional exchanges between brain and blood [1,2]. The BBB, initially observed in brain capillary beds and refined then to be held by brain microvessel endothelial cells (ECs) [3,4], was for a long time a concept whose demonstration and characterisation evolved and continues to evolve with technological advances. Its formation relies on a careful coordination of inducing factors originating from the cells of the ECs’ cellular microenvironment, namely (i) the brain pericytes (BPs) that share the same basement membrane as the ECs, (ii) the pedicellar extensions of the astrocytes that continuously surround the cerebral microvessels, and (iii) the neurons or neuronal progenitors in the perivascular brain parenchyma. All these cells, including ECs, form the neurovascular unit (NVU) [5]. Like its history, the cell–cell communication patterns between ECs and other cellular constituents of the NVU evolve during development according to a unique timeline and complementarity, the maintenance of which is altered by age and/or by central or peripheral pathological processes [6]. Furthermore, the evolution of techniques and the use of in vitro models have made the characterisation of new cell–cell communication vectors possible, such as extracellular vesicles (EVs) whose role in the induction and maintenance of the BBB phenotype remains to be fully characterised. The return to the history of the BBB proposed by this review will shed light on the first suggestions of the importance of cell–cell communications within the NVU to first explain the existence of this particular phenotype at the level of cerebral microvessels, then the emergence of these communication pathways during its development or barriergenesis, as well as during pathophysiological events.

## 2. The Blood–Brain Barrier, a Historical Concept Evolving with Technological Advances

### 2.1. Emergence of a Concept

**Figure 1:**
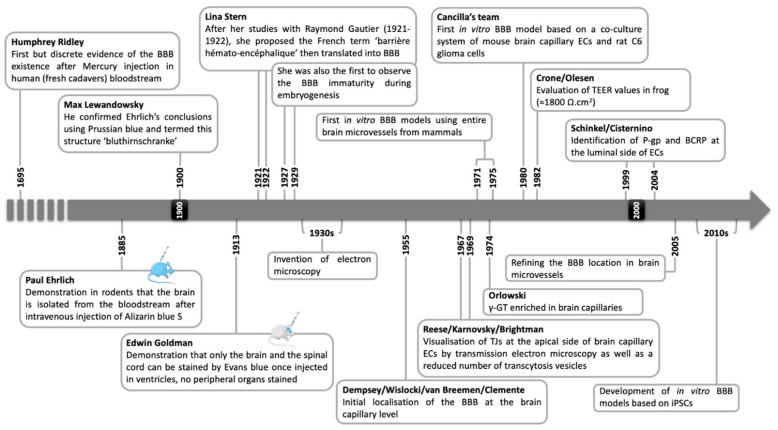
**The BBB through essential dates**. *Abbreviation: **BBB**: Blood–Brain Barrier, **BCRP**: Breast Cancer Resistance Protein, **ECs**: Endothelial Cells, **iPSCs**: inducible Pluripotent Stem Cells, **P-gp**: P-glycoprotein, **TEER**: TransEndothelial Electric Resistance, **γ-GT**: γ-GluramylTranspeptidase*.

Although the concept of the BBB emerged in the early 20th century, its history begins three centuries earlier (Figure 1). Indeed, the first observations of what can be considered as the BBB date back to the end of the 17th century thanks to the pioneering work of Humphrey Ridley. At that time, vivisection was already being carried out on so-called “inferior” animals or humans but was not ethical for religious reasons. Ridley advocated and practiced his observations and experiments on human subjects, either dead (fresh corpses) or dying. He was one of the first anatomists to study the human vasculature precisely by injection of coloured wax or mercury, the observation of smaller vessels such as capillaries and microvessels being easier using mercury. Above all, he was the first to demonstrate the important size and distribution of the cerebral vascular network, as well as the low permeability of small cerebral vessels in comparison with peripheral microvessels, although he did not fully understand the significance of this discovery [7].

It was not until almost 200 years later that this discovery resurfaced through experiments on rodent models by Paul Ehrlich in 1885. By injecting vital dyes such as Alizarin blue S into the peripheral vascular system of a rodent, he was able to demonstrate two things: (i) this dye diffused throughout the vascular tree and peripheral tissues, and (ii) neither the brain nor the cerebrospinal fluid (CSF) was stained. His conclusions were that the brain has a very low affinity for this dye, or that the high cell density in the brain does not allow its diffusion within the brain and CSF [8]. At least, this work suggested the existence of an interface separating the brain from the general circulation, which was postulated later by the work of Bield and Kraus in 1898 and Lewandowski in 1900. They demonstrated that the CNS was protected from circulating compounds such as cholic acid when administered intravenously, but provoked a central response once administered intraventricularly [9,10]. As Ehrlich earlier, Max Lewandowski conducted some experiments using Prussian blue to stain the bloodstream with the same conclusions and named this new concept “bluthirnschranke” in 1900, the German name for BBB. He shared the doubts of Ehrlich and the scientific community as to the concrete existence of this interface, especially since no tool could allow direct observation of what it might be [10].

Some years later, an Ehrlich’s student, Edwin Goldman, repeated between 1909 and 1913 Ehrlich’s previous experiment but chose to inject Trypan blue directly into the cerebral ventricles to target the CSF. His observations initially refuted those of Ehrlich insofar as the brain tissue and CSF were stained by Trypan blue. However, he observed that no peripheral organs were stained, demonstrating that the dye could not diffuse into the bloodstream [11]. This interface was therefore not only a barrier to the entry of circulating dyes, but also to the exit of dyes injected into the brain, i.e., an interface that isolated the brain from the rest of the organism in a bidirectional manner. Between 1918 and 1925, Lina Stern and Raymond Gautier carried out complementary works demonstrating that this interface (i) has little or no permeability for entry of many circulating substances or compounds into the brain compartment, and (ii) is more permissive for exit into the bloodstream of compounds administered into the brain [12,13]. Stern named this interface in 1922 “barrière hémato-encéphalique” in French or “hemato-encephalic barrier” in her German and Russian articles, which was then translated into blood–brain barrier (BBB). A few years later, Stern also described that the BBB was not mature during embryonic development and in newborn animals [14,15].

### 2.2. The Proofs of Concept

The existence of the BBB was no longer in doubt at the end of the 1920s, and it seemed to be located in the cerebral blood vessels. The findings at the time also stated that (i) a substance capable of entering the brain can be measured in the CSF and (ii) substance entry into the brain via the CSF is possible if it does not cross the BBB. In other words, and supported by Walter’s hypothesis, the CSF was the obligatory step in the passage of a blood compound into the brain, which imprecisely allowed the first mathematical modelling of diffusion of molecules between the blood and the CSF as existing at the cellular level [16,17,18]. The invention of the electron microscope in the 1930s and its use for cell imaging were crucial for the observation of the BBB and its distinction from the blood–CSF barrier. The first imaging studies of BBB date back to 1955 with the work of two teams using silver nitrate labelling for transmission electron microscopy. They then defined the location of the BBB in the cerebral microvessel network [19,20]. Later, by observing the obstruction of the perivascular space by the continuity of the astrocyte end-feet, some assumed that either this barrier was located at this level or that astrocytes were important in the construction of this barrier [21,22]. This hypothesis was fuelled by the observation that X-ray irradiation of simian brains caused significant damage to the astrocytic feet, which would be responsible for the entry of circulating dyes into the brain parenchyma [23]. In 1967, Reese and Karnovsky were the first to observe the BBB structural basis with an electron-dense zone in mouse brain capillary ECs using horseradish peroxidase. They highlighted the presence of apical junctions at the EC level and restricted transcytosis-related vesicles referred to as pinocytic vesicles at the cell surface. They concluded that the anatomic state of the BBB was in brain microvessel ECs, and not in astrocyte end-feet [24,25]. Reese continued his research and identified these junctions as tight junctions (TJs) and first determined the polarity of brain capillary ECs [26], and helped to highlight the low number of aspecific transport vesicles—macropinocytosis or micropinocytosis—at the apical and basolateral poles of ECs [26,27].

Given the complexity of studying the cerebral vascular network, as well as understanding the origin and mechanisms at work to build such a particular phenotype, the development of in vitro BBB models appeared to be essential. The first models based on the isolation of entire brain capillaries from different mammalian species shed light on the transport of amino acids [28,29,30] and their polarised transport, particularly for neutral amino acids [31,32,33,34], as well as the transport of soluble metabolites [35] and glucose [36,37,38]. γ-glutamyltranspeptidase (γ-GT), responsible for the proteolytic cleavage of peptides into amino acids, is localised in the CNS [39] and is enriched in brain capillaries [40]. Determining experiments led by Cancilla’s team using isolated microvessels and subsequent isolation of brain microvessel ECs from mice (the ME-2 cell line) have demonstrated that (i) the expression and activity of this enzyme is significant in these cells and (ii) both are induced in vitro in a co-culture system of ME-2 cells seeded on a microporous polycarbonate filter with C6 rat glioma cells [41,42]. This experimental approach is one of the first referenced as using an in vitro BBB model as we know it today and pointed out the importance of cell–cell communications between ECs and surrounding cells such as glial cells to induce or maintain the BBB properties.

Moreover, in line with the knowledge of the paracellular diffusion properties of ions for cells possessing TJs and the advancement of measurement techniques, pioneering work on measuring the transendothelial electric resistance (TEER) of the BBB was carried out in a batrachian model and revealed high TEER values of the order of 1800 Ω.cm^2^ [43]. These measurements have been obviously done in mammals and the overall in vitro BBB model developed since [44]. TEER measurement is now used routinely for in vitro evaluation of the BBB integrity but (i) is only reflecting the BBB permeability for ions, (ii) can differ according to the measurement and calculation methods, and (iii) is somehow experimenter-dependent [45]. It is therefore recommended to combine TEER evaluation with BBB permeability assays for integrity marker molecules such as small-molecular-weight dextran, Lucifer yellow, or sodium fluorescein [44].

### 2.3. To the Current View of the BBB Main Features

**Figure 2:**
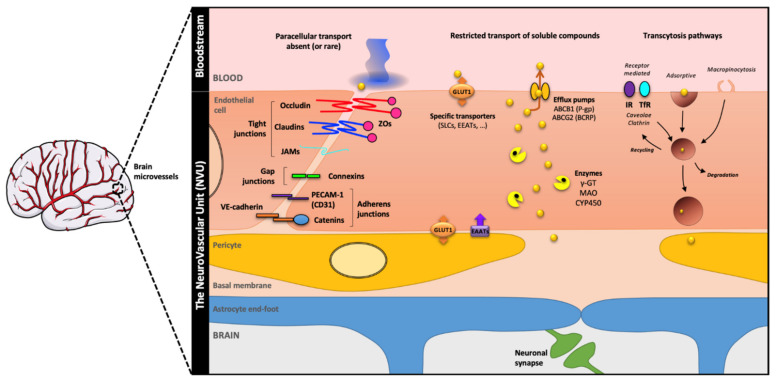
**A current overview of the BBB main features**. The brain microvessel endothelial cells (ECs) form the BBB that separates the brain and the blood and restricts the exchange between both compartments in order to preserve brain homeostasis. The BBB phenotype, which exhibits two main features referred to as physical and metabolic barriers, is the product of cell–cell communications between ECs and (i) brain pericytes, (ii) astrocytes via their pedicellar extensions, and (iii) progenitor and mature neurons. The physical barrier is defined as such due to (i) the presence of multiple junctional complexes such as TJs which connect ECs, composed of a specific protein complex including claudins, occludin, tricellulin, and zonula occludens (ZOs) proteins, and (ii) reduced transcytosis processes mainly led by adsorptive and receptor-mediated pathways. The metabolic barrier is linked to (i) the limitation of the free diffusion of small soluble compounds by the expression of degradative enzymes by ECs such as monoamine oxidase (MAO) or insulin-degrading enzyme (IDE), and (ii) the presence of efflux pumps belonging to the ABC transporters family which return undesirable molecules into the bloodstream such as xenobiotics. *Abbreviation: **ABC**: ATP Binding Cassette, **BBB**: Blood–Brain Barrier, **BCRP**: Breast Cancer Resistance Protein, **CYP450**: Cytochrome p450, **ECs**: Endothelial Cells, **EEATs**: Excitatory Amino acid Transporter 2, **GLUT1**: Glucose Transporter 1, **iPSCs**: inducible Pluripotent Stem Cells, **IR**: Insulin Receptor, **JAMs**: Junctional Adhesion Molecules, **MAO**: MonoAmine Oxidase, **PECAM-1**: Platelet Endothelial Cell Adhesion Molecule 1, **P-gp**: P-glycoprotein, **SLCs**: Solute Carriers, **TEER**: TransEndothelial Electric Resistance, **TfR**: Transferrin Receptor, **TJs**: Tight Junctions, **VE-cadherin**: vascular Endothelial-cadherin, **ZO**: Zonula Occludens, **γ-GT**: γ-GluramylTranspeptidase*.

Until the late 1980s, knowledge about the BBB could be summarised as (i) its physical properties based on TEM observations, (ii) its transport selectivity and polarity, and (iii) the action of γ-GT in restricting/redistributing the diffusion of circulating or brain compounds. However, the main molecular players responsible for these properties such as TJ proteins or efflux pumps are not yet identified, or at least the means of the time did not allow it. As Pardridge and colleagues reported in 1986, only a general Coomassie Blue protein profile comparing isolated human and bovine capillaries was known, which suggested that a 46 kDa protein might be part of the TJ composition [46]. The explosion of analytical tools in molecular biology and biochemistry made their separation, identification, and study possible. The main proteins belonging to the BBB phenotype are listed below (Figure 2, deciphered in more details elsewhere [1]).

The identification of BBB TJs proteins followed their study in various tissues with TJs and particularly the intestinal epithelium. Occludin [47] was identified first, followed by claudins [48,49] with a majority expression of claudin-5 in TJs of brain capillaries [50,51]. Occludin and Claudin-5 are two transmembrane integral proteins that form the intercellular link between two adjacent ECs. Tricellulin, identified in 2005, completes the composition of TJs at areas of tricellular contact [52]. TJs proteins are anchored to the actin cytoskeleton via Zonula Occludens (ZOs) proteins, mainly ZO-1 [53,54,55]. Basolateral adherens junctions (AJs) with cadherin-5 or vascular endothelial-cadherin (VE-cadherin) as main representative protein [56,57] and medial junctional adhesion molecules (JAMs) complete the junctional complexes exposed by brain capillary ECs and have been well described elsewhere [1,58,59,60]. Thus, also in connection with the low rate of non-specific transcytosis, all these junctional complexes drastically restrict the paracellular passage of soluble blood compounds, circulating microvesicles and cells such as circulating lymphocytes or macrophages, which can only cross the BBB in response to inflammation of the brain compartment [61,62,63,64].

The selectivity of BBB towards circulating compounds or brain metabolites is in close association with (i) the polarised expression of specific transporters such as solute carriers transporters (SLCs, [65]) such as the glucose transporter GLUT1 (SLC2A1) at EC basolateral side [66,67,68], and (ii) the presence of efflux pumps belonging to the family of ATP-binding Cassette (ABC) transporters [65,69]. Among these efflux pumps, the best known is P-glycoprotein (P-gp or ABCB1), identified in 1989 [70] and whose role and expression at the apical pole of ECs are conserved in all mammals [71]. The discovery of ABCG2 is related to the resistance of breast tumours to chemotherapeutic agents such as anthracyclines, hence its name Breast Cancer Resistance Protein (or BCRP) [72]. Its expression at the apical pole of brain microvessel ECs is also found in many animal species [73,74]. As mentioned before, the other identified and studied efflux pumps and the enzymes restricting the free diffusion of soluble compounds such as endothelin-1 (ECE-1) or monoamine oxidase (MAO) have been well described elsewhere [1].

As established during the pioneering work to identify the BBB concept and its validation as technology progresses, all the currently known characteristics of BBB are the result of cell–cell communication between ECs and the cells in their close microenvironment, namely BPs, astrocytes, and neurons [1,75]. This phenotypic induction that makes the BBB so special responds to a precise timing between all the cellular protagonists during brain development, after birth, and must be maintained throughout life to preserve brain homeostasis.

## 3. Cell–Cell Communications for the Establishment of the BBB during Embryogenesis

### 3.1. The Different Steps of the BBB Development or Barriergenesis

For a long time, the foetal BBB was described as primitive or immature, but recent developmental studies led on various species such as rodents and pigs show that it is functional before the birth [76]. Cerebral vascularisation begins two weeks after the onset of cerebral cortex development, i.e., at the 8th embryonic week (E8), from the hindbrain to the forebrain [77,78]. The establishment of the BBB starts at E12, and at this early stage, the endothelium already expresses some key proteins of the BBB phenotype. Indeed, claudin-5 and occludin, proteins of TJs, are present in the cytosol of ECs, and will be delivered to their functional site, i.e., the plasma membrane from E14. From E18, TJs are able to retain high molecular weight molecules, allowing restriction of paracellular passage. The barrier function of the BBB is optimal within a few weeks, with the structuring of the TJs similar to that of an adult BBB from E18 and whose complexity will be optimal after birth [79,80,81,82] as was initially described in Stern’s studies [14,15].

Some transporters are also expressed early in development, such as the glucose transporter GLUT1, expressed from E12 and whose complexity and distribution will be also optimal after birth. Its precocious expression can make it be referred to as a marker of the BBB development [82,83]. In addition, SLC transporter-type ion transporters are expressed at early embryonic stages and are functional in the developing brain [84].

The foetal BBB rapidly acquires its physical and metabolic barrier properties. The use of genetically modified mouse models has allowed the identification of certain proteins involved in the acquisition of this unique BBB phenotype such as the reduction of endothelial permeability, thanks to the role of the protein Major Facilitator Superfamily Domain Containing 2A (Mfsd2a). Indeed, as observed in in vivo experiments using Dextran as integrity marker, a loss of Mfsd2a expression during embryogenesis induces vascular leakage [85].

Transcytosis pathways are highly regulated at the BBB. LDL is transited across the BBB by endocytic vesicles after internalisation mediated by the LDL-specific receptor. In addition, these pathways are controlled by other components of the NVU, such as astrocytes that can modulate LDL transcytosis according to their lipid needs [86,87].

A study in rats showed an increase in protein expression of the iron transporter Tfr after birth, whereas the expression of the insulin receptor (IR) is constant throughout BBB development, thus meeting the needs of brain cells even in embryonic development [88,89].

Furthermore, an increase in P-gp and BCRP is observed as early as the first day after birth, accompanied by an increase in the amount of caveolin, a transcytosis protein involved in P-gp transport [88]. These observations are not available for all species, since an increase in P-gp before birth has been observed in monkeys, highlighting that P-gp expression would be dependent of the species studied and of the degree of maturation of brain compartment [90]. During embryogenesis, the development of transcytosis mechanisms within the human BBB remains poorly understood. Additional studies on peripheral vessels would allow a better understanding of the different stages of development of vesicular transport.

Finally, BBB construction starts at an early stage of embryogenesis allowing the establishment of a functional BBB early in development, and its maturation is completed after birth. This hierarchised process follows the cell–cell communications processes between ECs and its neighbouring cells that will constitute the future NVU.

### 3.2. Role of the NVU Components in Barriergenesis

**Figure 3:**
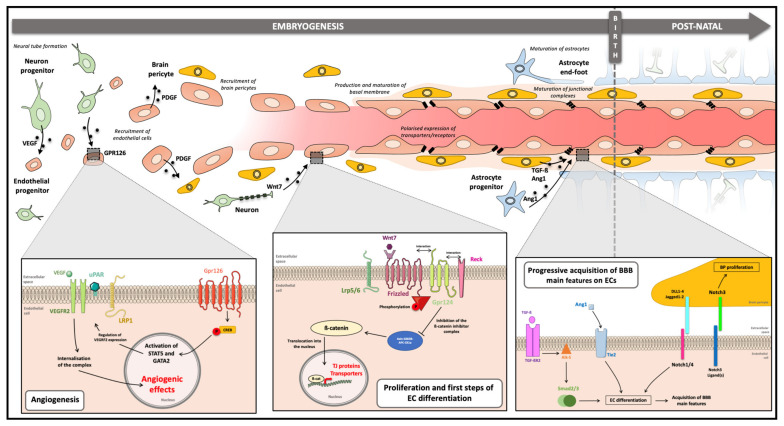
**Cell-cell communications during barriergenesis**. During embryogenesis, establishment of the BBB phenotype is possible through cell–cell communication processes between components of the NVU. Endothelial progenitors colonise the neural tube in response to the VEGF gradient secreted by neural progenitors, which promotes angiogenesis. The interaction of future BBB ECs with neurons activates several signalling pathways of which the most studied is the Wnt pathway. Thus, the ECs continue to proliferate, and the first steps of differentiation begin. Once established, future ECs meet BPs by secretion of PDGF; this cell–cell interaction is crucial for the differentiation of cells into mature BBB ECs and involves Notch and Smad-dependent signalling pathways. Establishment of the BBB continues until the birth and maturation of astrocytes. *Abbreviation: **Alk5**:Receptor protein serine/threonine kinase,****Ang1:****Angiopoietin 1, **APC**: Adenomatous polyposis coli, **BBB**: Blood–Brain Barrier, **CK1a**: Casein Kinase 1A, **CREB**: C-AMP Response Element-Binding Protein, **DLL**: Delta-Like Ligands, **EC**: Endothelial Cell, **GATA2**: GATA Binding Protein 2, **GPR**: G-Protein coupled Receptor, **GSK3β**: Glycogen Synthase Kinase 3 β, **Jagged**:Protein jagged-1, **LRP**: Lipoprotein Receptor-Related Protein, **Notch**:Neurogenic locus Notch protein, **PDGF**: Platelet Derived Growth Factor, **Reck**: Reversion-Inducting-Cysteine-Rich protein, **Smad******:****Mothers Against Decapentaplegic,****STAT5:****Signal Transducer and Activator of Transcription 5, **TGF-β**: Transforming Growth Factor β, **Tie 2**: Tyrosine kinase with immunoglobulin and EGF homology domain 2, **TJs**: Tight Junction, **uPAR**: urokinase Plasminogen Activator Surface Receptor, **VEGF**: Vascular Endothelial Growth Factor, **VEGFR2**: Vascular Endothelial Growth Factor Receptor 2, **Wnt7**: Wingless Int 7.*

In 1981, Stewart and Wiley highlighted the importance of cell–cell communications for the establishment of BBB main features. They demonstrated in ovo with quail’s eggs that a brain tissue graft was sufficient to establish the BBB phenotype in intestinal ECs [91]. The role of neural progenitors, BPs, and astrocytes in this process is summarised and figured below (Figure 3).

#### 3.2.1. Neuron Progenitors

During the cerebral vascularisation, endothelial progenitor cells (EPCs) migrate into the neuroectoderm following the gradient of a vascular endothelial growth factor (VEGF) secreted by neural progenitors. VEGF binds to the EC receptor VEGFR2 (flk-1/KDR), whose expression is modulated by the recently discovered receptor G Protein-coupled Receptor 126 (GPR126). The binding of the ligand to its receptor promotes a dimerisation and phosphorylation on a tyrosine kinase site that allows the recruitment of the urokinase plasminogen activator receptor (uPAR) by integrins β1 [92]. uPAR acts as an adaptor to bring the low-density lipoprotein receptor-related protein 1 (LRP1) within this complex to induce its internalisation necessary for the activation of several signalling pathways involved in cell proliferation, such as the MAPK/ERK pathway [92,93,94]. This angiogenesis process is based on the activation of several signalling pathways, the best known of which being the Wingless Int (Wnt) pathway. Canonical Wnt signalling is involved in the stimulation of target genes, including genes for BBB phenotype in ECs. After binding of the Wnt ligand—Wnt7a and b—secreted by neuronal precursors, a complex cellular cascade is activated involving Frizzled receptors that form a complex with LRP5/6 coreceptors. Recently, new proteins have been discovered in this protein binding process, the G protein-coupled receptor GRP124, and the Reversion-Inducting-Cysteine-Rich protein (Reck), both aiming to stabilise and activate the ligand-receptor complex [95,96,97]. This multi-protein complex promotes the stabilisation of ß-catenin, a protein that acts as a transcription factor in the cell nucleus. Thus, transcription of several genes required for the formation of TJs is promoted, such as gene coding for claudin-3 and claudin-5 proteins, and those involved in vesicular transport are inhibited Plasmalemma vesicle-associated protein (*PLVAP*) [98,99,100]. Moreover, the Wnt pathway promotes the expression of BCRP, an efflux pump present since embryogenesis [101], but also of PDGF-β (platelet-derived growth factor-β), a factor involved in the recruitment of another cell type, the BP [102].

#### 3.2.2. Brain Pericytes

Following the pro-angiogenic process initiated by neural progenitors, ECs recruit BPs, which play a pivotal role in the establishment of the BBB and whose recruitment coincides with the early stages of the appearance of the barrier phenotype during embryogenesis. The growth factor PDGF-β, secreted by ECs, binds to the PDGFR-B receptor expressed on the surface of BPs [103]. The BP coverage allows close contacts between ECs and BPs and the sharing of the same basement membrane [82]. Contact between BPs and ECs induces the secretion of TGF-β (transforming growth factor-β) by both cell types. TGF-β binds to its receptor TGF-βR2 which activates Smad pathway. The TGF-βR2 binds the receptor Alk5, which phosphorylate Smad2/3 proteins to recruit Smad4, and the formed complex acts as a transcription factor to promote genes involved in the formation of capillary network and endothelial basement membrane. Moreover, this pathway promotes pericyte and endothelial extracellular matrix formation, as well as the production of N-cadherin protein, an adherens junction protein that enhances pericyte adhesion [58,104,105]. The close interaction between ECs and BPs induces the formation of specialised junctions, called peg and socket, which allow the exchange of small molecules, such as growth factors between ECs and BPs [106,107,108,109].

Moreover, Notch signalling also plays an important role in the bidirectional communication between ECs and BPs. On the one hand, the Notch3 ligand expressed by ECs binds its receptor to BPs, which promotes BPs proliferation through a possible positive regulation of PDGFR-β. As reported in mouse models [110] or in Zebrafish [111], a deficiency or inhibition of Notch3 directly impacts the pericyte covering and thus the integrity of the BBB. On the other hand, Notch 1 and Notch 4 expressed by ECs interact with their ligands present on the surface of BPs, which stimulates a Smad4-dependent pathway, promoting EC proliferation [112,113,114]. The importance of Notch4 in the differentiation of rat cerebral microvessel ECs has also been reported [115]. Nevertheless, the elucidation of the role of Notch pathway in ECs remains not fully understood.

Finally, and despite a late interest within the BBB research community, BP is a major inducer of BBB at the embryonic level, participating in the loss of fenestrations, the structuring of TJs, and the restriction of endothelial vesicular transport. In addition, the pericyte secretes angiopoietin-1 (Ang-1) which binds to the endothelial tie2 receptor, thereby enhancing vascularisation and cell survival [116,117].

A loss of pericyte coverage coincides with an increase in endothelial permeability due to poor architecture of TJs, accompanied by cytoplasmic relocalisation of certain junctional proteins such as occludin and VE-cadherin, as well as an increase in specific vesicular transport, especially the caveolae-dependant transcytosis. The absence of BPs directly impacts the morphology and number of ECs, probably due to increased cellular stress [99,118,119]. In vitro analysis of the EC transcriptome after soloculture or coculture with BPs revealed a small number of genes involved in the establishment of the BBB phenotype, particularly a decrease in *Plvap* genes responsible of the fenestration of ECs [120]. These analyses are in agreement with the in vivo observations done by Daneman and colleagues [99].

#### 3.2.3. Astrocytes

In contrast to BPs and contrary to some historical hypothesis [21,22], astrocytes arrive later within the forming NVU. Contacts between ECs and astrocyte feet are not observed in the early stages of development; however, early contacts are thought to direct the fate of glial cells by inducing astrocytic properties and thus promote their differentiation [121,122]. Mature neural cells, whose role in the induction of the BBB phenotype is rather controversial, are present a few weeks after the start of neurogenesis during embryogenesis. In addition, a few in vitro studies have demonstrated the importance of astrocytes in the establishment of a tight and functional BBB. In the 1990s, the role of astrocytes was associated with the differentiation of endothelial precursors into ECs by secretion of factors influencing EC division and γ-GT expression [123]. Following these observations, the use of in vitro models based on co-culture between ECs and astrocytes were used and highlighted the role of astrocytes in (i) reducing endothelial permeability through a modulation of TJ proteins and in (ii) increasing endothelial electrical resistance [51,86,124,125]. A few years later, it was shown that coculture with astrocytes in vitro induced the expression of the efflux transporter P-gp, a key marker of the BBB phenotype [126,127]. Furthermore, astrocytic precursors are thought to enhance neuronal activity and stimulate the production of angiopoietin 1 by pericytes, thus improving barrier function [58]. However, the main limitation of the initial in vitro models is the use of adult brain ECs, and few if any studies have been able to experiment with foetal brain ECs due to the low number of cells developed at this stage [80]. Contrary to the used adult or mature ECs, endothelial progenitors from cord blood or deriving from induced Pluripotent Stem Cells (iPSCs) seem not to be reactive to astrocyte-secreted factors in terms of barriergenesis, although a decrease in endothelial monolayer permeability was observed in in vitro studies. However, these cells are sensitive to BPs from brain biopsies [128,129,130] or iPSC-derived BPs [131]. These arguments are in favour of a later and/or a leaker induction of the BBB main features by astrocytes as Daneman and colleagues exposed earlier [117].

However, the role of astrocytes would be clearer at the time of phenotype maturation through the activation of an important cellular pathway, the Sonic hedgehog pathway, involved in endothelial polarity [58,132]. Disruption or absence of this signalling induces a decrease in the expression of certain TJs proteins, thus inducing a more permeable barrier. This indicates that the long-term structuration of TJs is astrocyte-dependent [133,134].

As developed above, the establishment of the BBB, termed barriergenesis, is the result of finely regulated cell–cell communication processes within the forming NVU, and according to the latest works, BPs have a major role in this process. In concert, the future cellular components of the NVU will also allow the maturation and maintenance of the BBB phenotype on ECs after birth.

## 4. Maintenance of the BBB Phenotype through Cell–Cell Communications

Cell-cell communications between ECs and NVU cells are even more important for the maintenance and the maturation of the BBB main features after birth. Indeed, the BBB phenotype is reinforced, in particular by the expression of additional junctional proteins such as Zonula Occludens (ZOs), to anchor them to the actin cytoskeleton [51]. Thus, the whole NVU seems to adapt in order to maintain a mature barrier function and to cope with environmental changes such as pathological processes.

### 4.1. Role of the NVU Components in the BBB Maintenance

#### 4.1.1. Brain Pericytes

Having a major role in the induction of the BBB phenotype, it is not surprising that BPs have an indispensable role in its maintenance. Pericyte loss in adulthood has serious consequences for both microvessel morphology and the BBB main properties. Indeed, vascular density is decreased, and vessel diameter is increased by dilation, with the presence of microaneurysms. In addition, BPs are thought to play a role in controlling cerebral blood flow by modulating the contractility of smooth muscle cells that make up cerebral arterioles. Moreover, a loss of BPs contractility leads to dilation of cerebral microvessels [135].

From the first days after birth, BPs acquire a mature morphology and can reinforce the ECs phenotype. Pericytes have an important effect in restricting the transport of molecules across ECs. BP loss is associated with a disruption of TJs accompanied by a decreased expression of occludin, claudins and ZO-1. Electron microscopic observation of TJs in such conditions shows a disruption of their structural alignment, causing an increase in paracellular transport of molecules that is inversely proportional to the number of BPs [99,136]. However, BP loss does not impact the expression of GLUT1 transporters, and is neither associated with an inflammatory context, nor with the activation of immune cells in young subjects, a mechanism involved during pericyte loss in aging subjects. Furthermore, the increase in paracellular transport is not associated with the appearance of fenestration in ECs [99,105,118].

BPs also regulate EC transcytosis processes as they influence the expression of certain proteins, such as Mfsd2a or PLVAP [85]. In response to BP loss, ECs improve the secretion of adrenomedullin, a protein involved in barrier protection, suggesting a compensatory effect on the part of ECs. Moreover, some metalloproteinases such as Matrix Metalloproteinase 9 (MMP9) are also upregulated in ECs, provoking an alteration of the EC extracellular matrix [99,137].

As for barriergenesis, BPs have a central role in the maturation and maintenance of BBB properties, but also in orchestrating communications between the ECs and other components of the NVU. It has been argued that BPs promote contacts with the astrocyte end-feet and thus ensure the role of astrocytes in the maturation of the BBB phenotype [138].

#### 4.1.2. Astrocytes

Astrocyte progenitors differentiate into astrocytes during BBB maturation and continue to proliferate during the first three weeks after birth. Thus, the astrocytes are mature, and the BP–EC interactions are reinforced with the establishment of astrocyte end-feet [78]. The interaction between ECs and astrocytes is important in the regulation of capillary diameter and blood flow through a calcium-dependent cell signalling pathway [139]. The establishment of astrocyte end-feet enhances the basement membrane composition of ECs, thereby maintaining pericyte function and BBB properties [140]. Furthermore, the BBB becomes more complex with the establishment of aquaporins such as AQP4 that will be fully expressed in adulthood [141].

However, the role of astrocytes in maintaining the BBB main features remains controversial. Many studies have shown that the ablation of astrocyte end-feet does not impact the BBB phenotype and that the formation and maturation of TJs are astrocyte-independent processes [136,142]. Nevertheless, astrocyte loss correlates with the increased microvessel diameter and disruption of endothelial proliferation [140]. An increase in VEGF expression is observed in astrocytes following birth, while its expression decreases in neuronal cells, supporting the fact that astrocytes also act as intermediaries in the communication between ECs and neuronal cells as they build closer contacts together [143].

In the light of this knowledge, it would seem that astrocytes have a minor role in the induction and maintenance of BBB properties but are essential for the regulation of the BBB under pathological conditions or in response to external stimuli [144]. Indeed, as mentioned earlier in this review, astrocytes allow the expression of LDL receptors on the luminal membrane of ECs, and that when needed, the latter can regulate the transcytosis of LDL to the brain compartment [87].

#### 4.1.3. Neurons

Little is known about the role of neurons in the establishment and maintenance of the BBB phenotype. As previously stated, communication between the neuron and the EC may occur via the astrocyte, which is spatially closer to the EC. It has been shown that neuronal activity allows the improvement of vascular architecture after birth, notably through the Wnt signalling pathway. The different key proteins involved in this cascade are regulated, including G protein-coupled receptors such as GPR124, which acts as a neuronally specialised co-activator [1,145]. Disruption or abolition of this signalling, including frizzled receptors, leads to a loss of BBB phenotype [100,146] and a loss of the barrier integrity [99,118].

It has recently been reported that cellular communication between the different components of the NVU can be managed through the exchange of extracellular vesicles (EVs). EVs are cell-derived vesicles and divided into three main subgroups according to their size: large, medium, and small EVs [147,148]. Initially described as a means of eliminating cellular waste, EVs have been widely described for nearly 20 years as vectors of cell–cell communication since these vesicles can carry regulatory factors such as cytokines, soluble proteins, and nucleic acids (microRNA or miRNA, long non-coding DNAs) [149]. These EVs, particularly exosomes belonging to the small EV subgroup, are involved both in the regulation of physiological processes such as the maturation of peripheral ECs by pericyte small EVs [150], or in the dispersal and entry into pathological processes as widely described in the context of certain cancers (for reviews, see [5,149,151]).

A neuronal microRNA cargoed by neuron-derived small EVs, miR-132, has recently been studied for its role as a regulator of cadherin-5, a protein of adherens junctions. According to this study carried out in zebrafish, miR-132 inhibits eukaryotic elongation factor 2 kinase (eef2k), which is a protein with repressive action on cadherin 5 expression. Thus, overexpression of eef2k would have a deleterious effect on BBB phenotype, highlighting the important role of miR-132 in maintaining the BBB integrity [152,153].

### 4.2. The BBB Maintenance in Pathological Conditions

**Figure 4:**
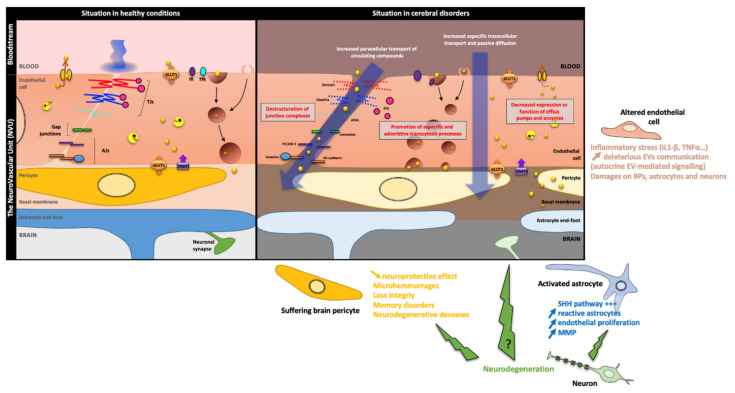
**Disturbances of cell–cell communication routes to maintain the BBB in pathological disorders.** Environmental changes due to age, external stimuli or certain neurodegenerative pathologies can alter the BBB over time. A loss of TJ sealing, a disruption of efflux pump activity and an increase in vesicular transport in ECs are therefore observed. Direct damage to ECs implies a deleterious inflammatory stress for the BBB, which will send some stress signals to other components of the NVU via EVs. To some extent, the BBB becomes more permeable to circulating molecules allowing the entry of external compounds and the extravasation of leukocytes into the CNS. The cell–cell communications between ECs and the different components of the NVU are altered. The damage on BP coverage leads to a decrease in the neuroprotective effect of these cells, leading to microhaemorrhages and neurodegenerative diseases. Activated astrocytes will promote endothelial proliferation with overactivation of the SHH signalling pathway, as well as a degradation of the endothelial basement membrane due to MMPs secretion. The direct role of neuron upon a neurodegenerative-dependent stress on the BBB features remains obscure. However, some studies suggest an indirect role based on altered cell–cell communication processes with both astrocytes and BPs leading to a BBB leakage. *Abbreviation: **AJs**: Adherens Junction, **BPs**: Brain Pericytes, **EEATs**: Excitatory Amino acid Transporter 2, **EV**: Extracellular Vesicle, **GLUT1**: Glucose Transporter 1, Cells, **IL1β**: Interleukin 1β, **IR**: Insulin Receptor, **JAMs**: Junctional Adhesion Molecules, **MMP**: Matrix MetalloProteinase, **PECAM-1**: Platelet Endothelial Cell Adhesion Molecule 1, **SHH**: Sonic HedgeHog, **TfR**: Transferrin Receptor, **TJs**: Tight Junctions, **TNFα**: Tumour Necrosis Factor α **VE-cadherin**: Vascular Endothelial-cadherin, **ZO**: Zonula Occludens, **γ-GT**: γ-GluramylTranspeptidase*.

Throughout life, the BBB has the capacity to adapt to environmental change and external stimuli to ensure the maintenance of the barrier phenotype and preserve brain homeostasis. However, and particularly with age, the BBB can face the development of certain pathologies that eventually lead to its alteration. It has been reported that in a pathological context, the BBB loses the tightness of the TJs which are downregulated, and an increase in vesicular transport in ECs is observed. ECs will increase the expression of certain adhesion molecules, such as Intercellular Adhesion Molecule-1 (ICAM-1), allowing leukocyte extravasation into the central nervous system [64]. These events are linked to a progressive loss/modulation of cell–cell communications within the NVU, leading to a progressive BBB leakage and therefore a destabilised brain homeostasis conducive to pathophysiological processes [137,144] (Figure 4).

#### 4.2.1. Brain Pericytes

Since BPs are major contributors to the establishment and maintenance of the BBB, it is not surprising that most neurological diseases are associated with BP dysfunction. Loss of pericytes at embryological stages is associated with cerebral microhaemorrhages, preventing the proper development of the embryo [154]. It is also a process found during erythrocyte loss in old age, causing a loss of vascular integrity. Various pathologies are therefore at the origin of pericyte dysfunction, which leads to a disruption of the barrier function. A study performed in 2012 focused on porcine stress syndrome, which is a disease homologous to malignant hyperthermia found in humans, demonstrated that the use of BPs from pigs with this syndrome significantly increased the permeability to BBB molecules in vitro and thus deteriorated its physical barrier function. The secretome of BPs has therefore a primordial role in the maintenance of the BBB phenotype [155]. In addition, BPs have a neuroprotective action by secreting pleiotrophin, which is a neurotrophic growth factor [155].

Damage to BPs weakens the maintenance of the BBB phenotype by the cells, as reported in a recent study highlighting the importance of maintaining postnatal expression by BPs of a target gene of the Notch pathway, the transcription factor Recombining Binding Protein Suppressor of Hairless (RBPJ). Although the effects described are not related to a direct alteration of the Notch pathway, the silencing of the *Rbpj* gene in BPs leads to (i) a change in the expression pattern of “pericyte-specific” proteins such as PDGFRβ, (ii) a change in basement membrane composition, (iii) a significant secretion of the *Rbpj* gene from the pericyte, (iv) a significant secretion of TGF-β inducing in particular the activation of Smad in ECs, and (v) the inhibition of the microvascular expression of the neuropilin-1 (Nrp1), co-repressor of the VEGF pathway, which is accompanied by a lifting of the inhibition of the phosphorylation of Smad2/3. This alteration in RBPJ expression in BPs promotes the proliferation of ECs through TGFß/VEGF pathways as during angiogenesis, and mimics the vascular damage observed during neurodegenerative processes without ablation of BPs [156].

The loss or the ablation of BPs coverage rapidly leads to neuronal cell damage and therefore degeneration [99,105,157]. In neurodegenerative diseases such as Alzheimer’s disease (AD), the consequences of pericyte loss have been widely described. Decreased clearance of β-amyloid (Aβ) peptide from the blood compartment induces accumulation of Aβ peptides in the brain compartment, and this accumulation enhances pericyte loss, which in the long term leads to BBB rupture [158]. However, a 2010 study showed that the depletion of BPs did not lead to learning disorders in young mice, whereas 15-month-old mice showed spatial memory disorders [136]. These different studies support the importance of BPs within the NVU. At different stages of development, pericyte loss is deleterious and is often associated with a variety of pathologies, particularly in aging with neurodegenerative diseases.

#### 4.2.2. Neurons

There are very few studies on the loss of communication between neurons and ECs in a pathological context. Nevertheless, in the context of AD, neuronal damage directly affects ECs, and this damage has been described in the very short term in the disease process [6]. Moreover, a loss of contact between neurons and ECs has been observed in the context of Parkinson’s disease, resulting in a loss of communication between these two entities [60].

The direct link between neuronal damage and BBB embrittlement remains difficult to characterise, as this link is associated with damage caused by neurons on other brain cell types, mainly astrocytes.

#### 4.2.3. Astrocytes

While the role of astrocytes is quite controversial under physiological conditions, there is ample evidence that they are important in the process of neuronal protection and barrier repair. Astrocytes are true intermediates between nerve cells and ECs in communication. A recent study also showed a deleterious effect of age on the BBB homeostasis through modifications on glial cells, particularly microglia and astrocytes [159], highlighting that all that alter the NVU components disrupt or at least modify the cell–cell communication pathways within the NVU to maintain the BBB phenotype. During brain injury, an inflammatory context is set up, inducing an overactivation of the Sonic hedgehog pathway, a pathway involved in development and which allows the cell division of stem cells, thus repairing the BBB [133,142].

The astrocyte, which has in a physiological condition a protective role of the BBB phenotype, has an antagonistic role in pathological situations. In AD, a breakdown of the BBB is observed in the early stages of the disease, and this breakdown is one of the major causes of cognitive decline. The accumulation of Aβ peptide, a major phenomenon in this pathology, induces the accumulation of reactive astrocytes. Thus, reactive astrocytes secrete factors promoting endothelial proliferation, such as VEGF, but also induce the expression of MMPs, thus altering the integrity of the BBB [160]. The loss or modification of cell–cell communication between astrocyte and BPs leads to the activation of cyclophilin-A in BPs, which promotes MMP9 expression. This altered communication within the NVU indirectly provokes TJs progressive disruption as well as a destabilisation of the basement membrane [161].

The cellular environment can also be disrupted by the development of cancer, particularly gliomas. Glioma cells communicate with their environment in order to adapt the conditions for their development. The exchange of EVs is used by these cells to transport several microRNAs, including miR-9-5P, which has an action on angiogenesis of the ECs [162]. In addition, the establishment of an inflammatory context can be deleterious to ECs since some signalling pathways are modulated, such as the RXR-α (Retinoid X receptor)-dependent pathway. The latter is inhibited when an inflammatory context is set up by contact with cytokines, such as Interleukin-1β (IL-1β) or Tumour Necrosis Factor-α (TNF-α), which are secreted during external aggression or in the event of pathology. Thus, certain BBB transporters are downregulated, affecting brain homeostasis [117]. The exchanges of EVs between the different components of the NVU would also play a role in senescence. These vesicles carry different cellular messengers and would allow the exchange of factors such as cytokines, including the Senescence-Associated Secretory Phenotype (SASP), which would have a deleterious action on ECs cell functions [163].

#### 4.2.4. Endothelium Damage

The damage done to the NVU components undoubtedly has visible consequences on the ECs and consequently on the BBB main features. However, damage to the endothelium can also cause changes in cell–cell communication within the NVU. This postulate comes from in vitro observations of the bidirectional interaction between cells in the construction of BBB phenotype, in particular the impact of ECs on BBBs when these cells are placed in co-culture. A study conducted by Dubey’s team showed the effect of ECs on the transcriptional expression and a decrease in the secretion of certain pro-inflammatory cytokines by BPs, notably growth-regulated alpha protein (CXCL1), 5, 8, and 10, or interleukins such as IL-1β [164]. The secretion of ILs, together with a significant increase in the expression of adhesion molecules such as ICAM-1 on the surface of ECs observed in the pathological context, promotes the infiltration of macrophages and the entry of circulating lymphocytes into the brain compartment [61,62,63,64,165,166]. Moreover, very few studies have demonstrated outside the context of cerebral ischaemia the consequences of endothelial damage on BPs, or astrocytes, with most focusing primarily on the damage to BBB main features. Romero’s team recently demonstrated in vitro that TNF-α-induced inflammatory stress at the endothelial level could be communicated to healthy ECs via EVs [167], which could also be communicated to other cell types in the NVU, but this remains to be demonstrated.

## 5. Conclusions and Perspectives

From its origin to its senescence, the BBB is the result of complex and finely regulated cell–cell communication pathways so that, in concert, the components of the NVU maintain the stability of the BBB and thus brain homeostasis. In addition to the expected and well-described cytokines and other soluble factors as vectors of cell–cell communication, work on the role of EVs and particularly small EVs in the maintenance and (dys)regulation of the BBB provides a new perspective and complexity in these exchanges. Thus, modulating these pathways as a preventive measure or as part of therapeutic approaches seems to be of importance to ensure the proper functioning of this vital barrier, especially in pathological conditions. As a recent example, a study performed in mice highlighted the promising role of the inhibitor of metalloproteinase-1 (TIMP) to protect the BBB by interacting with CD63 and integrin-1β to activate FAK/RhoA signalling, leading to EC structural stabilisation [168]. Moreover, the use of cargoes to address the CNS or the NVU cells such as nanopeptides, NPs, or EVs are in vogue in this quest for brain-targeted therapeutic solutions. A recent example in mice showed that intravenous injection of nanopeptides carrying a small interference RNA (siRNA) against β-secretase-1 (BACE-1) produced the expected brain response without side effects such as cytotoxicity or inflammation [169]. However, the issue of their low permeability for BBB remains problematic (for reviews, see [2,5]). Some strategies to increase the passage of these cargoes have been initiated, among them the use of Simvastatin to promote the expression and functionality of the LRP1 receptor, thus being able to optimise the transcytosis of circulating NPs through ECs [170]. However, given the complexity of these pathways, deciphering and deepening research to better control the plausible point of pharmacological intervention remains a challenge and presents new openings for the future.
